# Exploring Immune Development in Infants With Moderate to Severe Atopic Dermatitis

**DOI:** 10.3389/fimmu.2018.00630

**Published:** 2018-03-29

**Authors:** Lies Hulshof, Saskia A. Overbeek, Anne L. Wyllie, Mei Ling J. N. Chu, Debby Bogaert, Wilco de Jager, Leon M. J. Knippels, Elisabeth A. M. Sanders, Wim M. C. van Aalderen, Johan Garssen, Belinda van’t Land, Aline B. Sprikkelman, The Clinical Study Group, A. Blauw

**Affiliations:** ^1^Emma Children’s Hospital Academic Medical Centre, Department of Paediatric Respiratory Medicine and Allergy, University of Amsterdam, Amsterdam, Netherlands; ^2^Faculty of Science, Utrecht Institute for Pharmaceutical Sciences, Utrecht University, Utrecht, Netherlands; ^3^Nutricia Research, Utrecht, Netherlands; ^4^Department of Paediatric Immunology and Infectious Diseases, University Medical Centre Utrecht, Wilhelmina Children’s Hospital, Utrecht, Netherlands; ^5^Laboratory of Translational Immunology, Department of Paediatric Immunology, University Medical Centre Utrecht, Utrecht, Netherlands; ^6^Department of Paediatric Pulmonology and Paediatric Allergology, University of Groningen, University Medical Centre Groningen, Beatrix Children’s Hospital, Groningen, Netherlands

**Keywords:** atopic dermatitis, atopic dermatitis severity, chemokine profiles, dietary intervention, infants, T helper cell type 2/T helper cell type 1 response

## Abstract

**Background:**

Atopic dermatitis (AD) is the most common chronic inflammatory skin disease in infancy with a complex pathology. In adults, the clinical severity of AD has been associated with increases in T helper cell type (Th) 2, Th22, and Th17 serum markers, including high levels of CC chemokine ligand (CCL) 17 and CCL22 chemokines.

**Objective:**

To explore the possible association between serum chemokine levels and AD severity in infants with moderate-to-severe AD and elevated immunoglobulin E (IgE).

**Subjects and methods:**

Serum samples (*n* = 41) obtained from a randomized, double-blind, and clinical dietary intervention study were used to study biomarkers in infants with AD. Baseline- and post-intervention samples (4 months) were used, six chemokines and nine ratios thereof were analyzed using Luminex and correlated to AD severity. In the initial study, the infants were randomized to receive extensively hydrolyzed whey-based formula without (control) or with short-chain galacto-oligosaccharides/long-chain fructo-oligosaccharides (9:1) and *Bifidobacterium breve* M-16V (active).

**Results:**

31 Infants up to 11 months of age, with an objective-SCORAD score (oSCORAD) ≥ 20 and elevated total-IgE and/or specific-IgE levels were included. In time, the median oSCORAD decreased in both groups by −8 (control, *p* < 0.05; active, *p* < 0.01). Irrespective of dietary intervention, several changes in Th2 chemokines (CCL17 and CCL22), inflammatory chemokine (CCL20), and the Th1 chemokine, CXC chemokine ligand (CXCL) 9, were detected over time. Overall CCL17 correlated to oSCORAD (*r* = 0.446, *p* < 0.01). After 4 months of dietary intervention, CXCL9 was higher (*p* < 0.01) in the active group compared with control [active, 2.33 (1.99–2.89); controls, 1.95 (1.77–2.43) log 10 median (range)]. In addition, a reduction in Th2/Th1 chemokine ratios for CCL17/CXCL9, CCL22/CXCL9, CCL20/CXCL10, and CCL20/CXCL11 was detected associated with the active intervention.

**Conclusion:**

While this study is small and exploratory in nature, these data contribute to immune biomarker profiling and understanding of AD in infants.

## Introduction

### Atopic Dermatitis (AD) in Infancy

The natural course of atopic manifestations, also described as the atopic march, refers to AD and food sensitization during infancy and the development of allergic rhinitis or allergic asthma later in life (1, 2). AD, also known as allergic eczema, is the most common chronic inflammatory skin disease in infancy and childhood. The pathophysiology of AD is complex and involves a compromised skin barrier function, which allows penetration of environmental factors (including irritants, allergens, and bacteria) leading to inflammation and allergic sensitization (3, 4).

Upon cellular damage, keratinocytes react by releasing pro-inflammatory mediators and chemokines to attract leukocytes which follow a chemokine gradient to the site of inflammation (5, 6). Chemokine production at site of inflammation leads to leukocyte migration. Several chemokines [classically characterized within a T helper cell type (Th) 1-type of response: CXC chemokine ligand (CXCL) 9, CXCL10, and CXCL11; Th2 type of response: CC chemokine ligand (CCL) 17 and CCL22; and for inflammation: CCL20] have been suggested to be involved in development of AD (7). In adults, the clinical severity of AD has been associated with increases in Th2, Th22, and Th17-type of immune markers.

Only limited studies regarding the pathology of early pediatric AD are available and therefore only associations of disease activity to a few serum biomarkers [i.e., CCL17, CCL22, CCL27, and immunoglobulin E (IgE)] are known in infants (8–10). Serum levels of CCL17 detected in children with AD are higher than in healthy controls (11). In addition, significant correlations have been found between CCL17 and CCL22 and AD severity in infants as well as in adults (12, 13). Recently, immune activation in non-lesional skin in pediatric patients with AD has been detected (8). Alterations in skin-derived immunity and consequently the skin barrier function may occur during the early-onset phase of AD. For instance, Th2 (IL13, IL31, and CCL17), Th22 (IL22 and S100As), and some Th1-skewing mediators [interferon (IFN) γ and CXCL10] have been detected in the skin (8). A Th2 type of immunity classically leads to production of interleukins IL4, IL5, and IL13, which may locally induce IgE and eosinophilic activation, which collectively leads to allergic as well as atopic disease manifestations (14). Therefore, it is of interest to study the development of Th2/Th1 chemokine ratios in association to the severity of AD and the impact of specific dietary intervention.

### Dietary Intervention and AD

Evidence suggests that the microbiome of both the lung and gut are involved in the pathogenesis of asthma and allergy (15). Notably, recent studies have demonstrated essential differences between the microbiome profiles of the lungs from healthy and asthmatic subjects (16). It has been suggested that microbial modulations may induce beneficial effects in AD management, by decreasing the Th2-type response and increasing the Th1-type response (17, 18). In a dietary intervention study, a specific synbiotic mixture of prebiotic short-chain galacto-oligosaccharides (scGOS), long-chain fructo-oligosaccharides (lcFOS), and a probiotic strain *Bifidobacterium breve* M-16V resulted in significant improvement in IgE-associated AD severity in infants (19), leading to the prevention of asthma-like symptoms and asthma medication use at the 1-year follow-up (20).

Various pro-inflammatory mediators are released by mast cells upon activation and trigger allergic responses such as asthma, allergic rhinitis, and AD. In addition, anti-inflammatory and direct, microbiota independent, immune-modulatory properties of scGOS/lcFOS mixtures on human monocyte derived dendritic cells (DCs) have been suggested to induce regulatory T cells, which represents a way to induce maintenance of intestinal homeostasis (21).

Galectins are defined as lectins having both galactose-binding ability and specific regulatory amino-acid sequences. More specifically, galectin-9 is known to bind IgE, suppress IgE–antigen complex formation and, hence prevents pro-inflammatory mediator release from mast cells (22). In addition, galectin-9 expression by intestinal epithelial cells and serum galectin-9 levels has been shown to correlate with reduced acute allergic skin reaction and mast cell degranulation (23). A recent published *in vivo* study showed that the dietary intervention with prebiotics and *B. breve* M16-V enhances galectin-9 and suppresses allergic symptoms of OVA-allergic mice. This is associated with a reduced Th2 activation and increased Tregs frequency in these mice (24). This leads to the conclusion that dietary intervention may be associated with decreased maturation and activation of DCs, changing immune responsiveness (25). Therefore, in this study, we explored the immunological mechanisms behind the development of AD in infants with moderate-to-severe AD and elevated IgE levels.

## Subjects and Methods

### Subjects

Infants, aged 0–11 months, were recruited (from 2012 to 2014) at the pediatric and dermatology outpatient clinic in a tertiary referral center, Academic Medical Centre of Amsterdam. The objective SCORing Atopic Dermatitis score (oSCORAD) of ≥20 points, corresponding with moderate-to-severe AD, was required for enrollment in the study. In addition, elevated total and/or specific IgE levels were also required for inclusion. The cut-off value for elevated total IgE was considered as >5 kU/L at age <3 months and >15 kU/L at age >3 months. Specific IgE against aero-allergens or food allergens were considered elevated if >0.35 kU/L (19).

The use of topical corticosteroids was allowed under the guidance of the infants’ own general practitioner or dermatologist. However, it was recommended to adhere to a very strict policy with regards to the prescription of topical steroids. Topical steroid use was also permitted during the intervention period when the topical steroids where already prescribed before start, as it is unethical to withhold infants of AD treatment. Infants with severe cow’s milk allergy (history of anaphylactic reaction) were excluded. Infants treated with systemic corticosteroids, antibiotics or anti-mycotics 4 weeks, or antihistamines 48 h before enrollment were excluded. Infants with severe medical problems other than allergies were also ineligible for study inclusion. Infants with an expected daily intake of minimal 500 mL infant formula per day were eligible. Following enrollment, partial breastfeeding was allowed, and infants were required to drink ≥500 mL/day of the study infant formula.

### Study Design

A randomized double–blind clinical intervention study was conducted. All infants were randomized to receive a newly developed extensively hydrolyzed whey-based infant formula (eHF; Nutricia, Cuijk, the Netherlands) without (control group) or with prebiotic scGOS, long-chain fructo-oligosaccharides (lcFOS) and the probiotic strain *B. breve* M-16V (active group) for 4 months. This specific mixture consisted of 90% scGOS, 10% lcFOS (0.8 g/100 mL), and *B. breve* M-16V at a dose of 1.0 × 10^8^ cfu/g (Morinaga Milk Industry Co., Ltd., Tokyo, Japan).

Hospital visits were scheduled at screening, baseline (week 0) and after dietary intervention (4 months later). Gestational age, medical history, intake of infant formula, the SCORAD score (26), and blood samples were obtained at screening to investigate eligibility for randomization.

Biological samples were obtained before randomization (baseline) and at the end of intervention (4 months later), and oSCORAD score was assessed. During the study, parents maintained a diary to record daily intake of study product, fecal consistency, and inter-current illness.

### SCORAD Score

The most used and validated severity scale for AD is the SCORAD score, developed by the European Task Force on Atopic Dermatitis (ETFAD) (26) and is still one of the used measurements in AD trials by The Harmonising Outcome Measures for Eczema initiative (27). However, subjective symptoms, such as pruritus and sleep disturbance, are estimated by parents, which can be highly subjective. Therefore, the ETFAD modified SCORAD score (A/5 + 7/2) calculated by excluding the subjective symptoms (oSCORAD) was used in this study (28, 29). AD severity was classified as follows: oSCORAD 0–14 points mild AD, 15–40 points moderate AD, and >40 points severe AD (29).

### Blood Samples

Serum samples were obtained at screening and following 4 months of dietary intervention. Sample aliquots were stored at −80°C until analysis. Specific-IgE levels (total IgE, specific IgE against aero-allergens and food allergens) were tested with standardized methods using the CAP System FEIA (Phadia Diagnostics, Uppsala, Sweden). Using an in-house developed and validated (ISO9001 certified) multiplex immunoassay (Laboratory of Translational Immunology, University Medical Centre Utrecht) based on Luminex technology (xMAP, Luminex, Austin, TX, USA), the 90-plex assay was performed as described previously (30–32). Knowing the role of chemokines within AD pathology, specifically six chemokines (CCL17, CCL20, CCL22, CXCL9, CXCL10, and CXCL11) and nine chemokine Th2/Th1 ratios (CCL17–CCL20–CCL22 and CXCL9–CXCL10–CXCL11) were correlated to AD severity. After preabsorption with hetero-block (Omega Biologicals, Bozeman, MT, USA), acquisition was performed using Biorad FlexMAP3D (Biorad Laboratories, Hercules, CA, USA) in combination with xPONENT software version 4.2 (Luminex). Data were analyzed by five-parametric curve fitting using Bio-Plex Manager software, version 6.1.1 (Biorad).

### Initial Primary Study Outcome

The initial primary study outcome was change in severity of AD (oSCORAD) after 4 months of dietary intervention. However, the dietary intervention study was ended prematurely due to limited recruitment of this specific study population (AD in infants with elevated IgE levels). The specific requirements for study inclusion, however, did allow us to explore associations of AD severity and chemokine profiles in this unique population of infants with AD and elevated IgE levels.

### Statistical Analysis

Statistical analyses were performed using SPSS version 24 (IBM SPSS Statistics for Windows, Armonk, NY, USA) and GraphPad prism^®^ version 7.0 (GraphPad Software, La Jolla, CA, USA), and/or SAS^®^ Enterprise Guide 4.3 or higher (SAS Institute Inc., Cary, NC, USA). Statistical analyses of the exploratory measurements were conducted using non-parametric testing, with Wilcoxon matched-pairs signed rank test (W-test) for comparison within groups and with the Mann–Whitney *U* test (MWU) for non-paired, non-parametric comparison between groups. In addition, a two-tailed non-parametric Spearman correlation analysis was used to determine the overall associations between biomarkers as well as with clinical severity of AD (oSCORAD). Due to exploratory nature, differences were considered significant with a *p*-value of <0.05.

## Results

### Infant Characteristics

**Figure 1:**
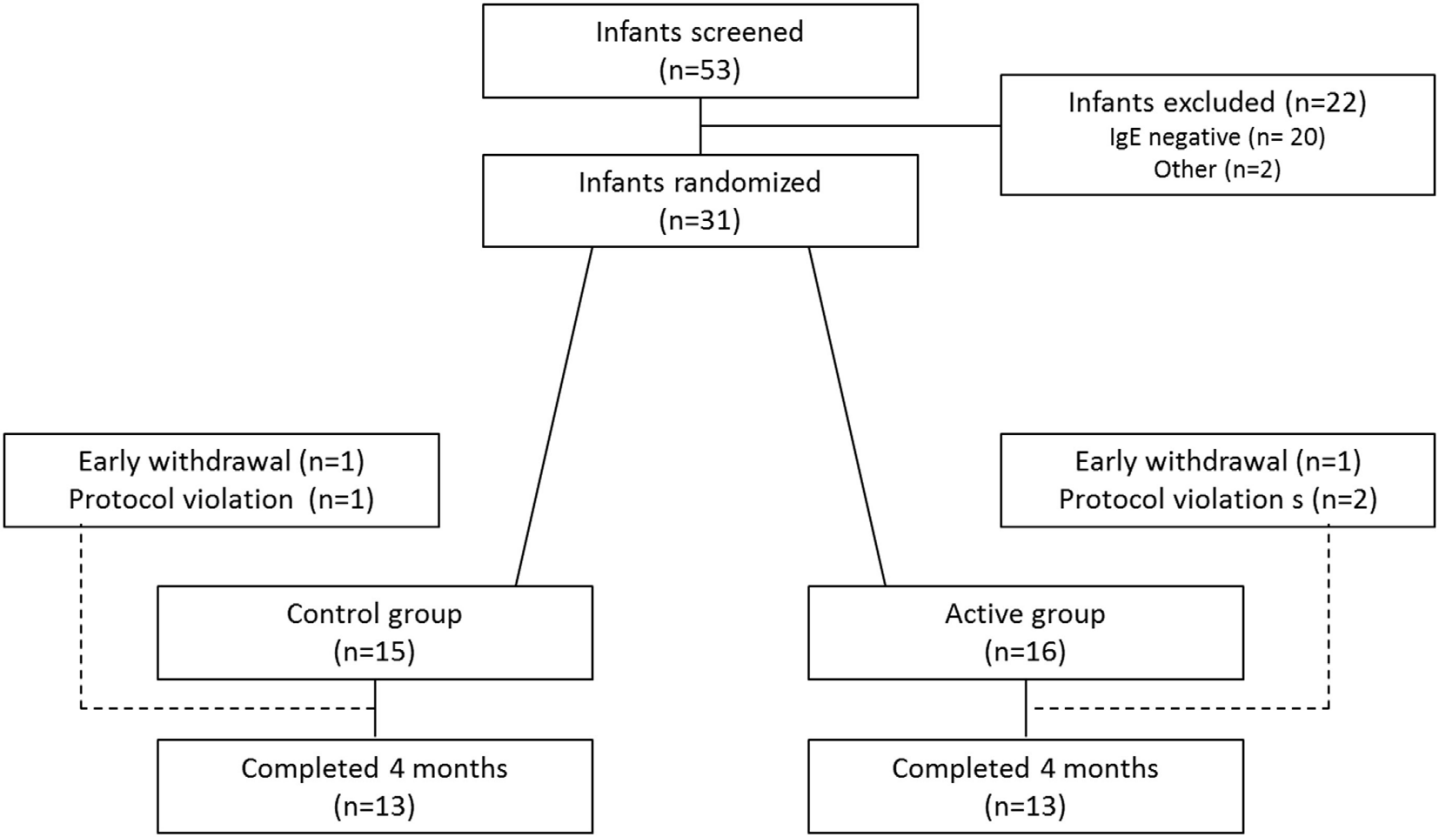
Flowchart of study inclusion and exclusion.

Overall, 53 infants with moderate-to-severe AD were screened. Of these, 31 infants had elevated IgE levels and were therefore considered eligible for randomization. In total, 26 infants completed the study (Figure 1). Baseline characteristics (*n* = 31) are shown in Table 1.

**Table 1:** Baseline characteristics of the clinical study (*n* = 31).

Characteristic	Control group (*n* = 15)	Active group (*n* = 16)	*p* Values
Male, *n* (%)	9 (60.0)	11 (68.8)	0.72
Age (months)			
Mean (±SD)	6.5 (1.8)	7.4 (2.4)	
Median (Q1–Q3)	6 (6–7)	8 (6–10)	0.15
Range	4–10	2–11	
Ethnicity, *n* (%)			0.82
Caucasian/White	11 (73.3)	13 (81.25)	
African/Black	1 (6.7)	0	
Asian	1 (6.7)	0	
Combination of above or other	2 (13.3)	3 (13.3)	
Gestational age (mean ± SD, weeks)	39.8 (1.2)	39.4 (1.8)	0.71
Birth weight (mean ± SD, g)	3,538.0 (428.6)	3,820.3 (718.2)	0.14
Delivery by cesarean section, *n* (%)	1 (6.7)	3 (18.8)	0.60
Length-for-age (mean ± SD, *z*-scores)	−0.4667 (0.9155)	0.3125 (0.9465)	**0.02**
Weight-for-age (mean ± SD, *z*-scores)	−0.5333 (0.7432)	0.125 (0.8062)	**0.03**
Head circumference-for-age (mean ± SD, *z*-scores)	−0.2143 (1.369)	0.0625 (0.7719)	0.32
Started with breastfeeding, *n*	13	14	
Duration of exclusive breast feeding (mean ± SD, months)	1.5 (2.1)	3.0 (3.1)	0.14
Time of first introduction cow’s milk protein (mean ± SD, months)	1.5 (2.1)	3.9 (4.3)	0.10
Time of first solid food introduction (mean ± SD, months)	5.3 (1.1)	5.2 (1.2)	0.86
oSCORAD score (mean ± SD)	26.53 (4.307)	24.94 (5.221)	0.22
Any topical corticosteroid used			
Yes, *n* (%)	14 (93.3)	15 (93.8)	1.00
Hydrocortisone acetate 1%, *n*	12	14	0.65
Triamcinolone acetonide 0.1%, *n*	5	4	0.70
Mometasone furoate 0.1%, *n*	1	2	1.00
Total immunoglobulin E (IgE)			
log 10 Mean (SD)	1.761 (0.4890)	1.562 (0.3508)	0.11
Total IgE log 10 min–max	1.000–2.963	0.8451–2.332	
Sensitization to aero-allergens			0.24
Negative, *n* (%)	7 (46.7)	11 (68.8)	
Positive, *n* (%)	6 (40.0)	3 (18.8)	
Missing, *n* (%)	2 (13.3)	2 (12.5)	
Sensitization to food allergens			0.65
Negative, *n* (%)	3 (20.0)	2 (12.5)	
Positive, *n* (%)	12 (80.0)	14 (87.5)	
Parental allergies: at least 1 positive allergic manifestations			
Mother (total 31), *n* (%)	6 (40)	6 (37.5)	1.00
Father (total 26), *n* (%)	7 (58.3)	7 (50)	0.71
Siblings in household (blood related)			
None	10 (66.7)	9 (56.3)	0.72
1	2 (13.3)	5 (31.3)	0.39
2 or more	3 (20)	2 (12.6)	0.65
Day care attendance [yes, *n* (%)]	11 (73.3)	12 (75.0)	1.00
Exposure to smoking in household [yes, *n* (%)]	5 (33.3)	4 (25.0)	0.70

The infants were well balanced over the study groups with respect to the baseline characteristics; only significant differences for length and weight were observed; infants in the control group were smaller in respect to length, weight. At baseline, the length-for-age *z*-scores for infants in the control group were significantly lower (*p* < 0.05) than the active group [mean (SD) −0.4667 (0.9155) versus 0.3125 (0.9465), respectively]. The weight-for-age *z*-scores were also significantly lower (*p* < 0.05) in the control group than the active group with a mean (SD) of −0.5333 (0.7432) and 0.125 (0.8062), respectively.

Atopic dermatitis severity was classified as moderate-to-severe with a median oSCORAD of 23 (Q1–Q3 = 21–27). All infants were born at term with a median gestational age of 39 weeks, and only 12.9% of the infants were born *via* cesarean section. The mean age of infants at the time of inclusion was 7 months (median 6, range 2–11 months). The male/female ratio was approximately 2:1 in both study groups. The majority (80%) of infants were of Caucasian descent. At baseline, total IgE was comparable in both groups. In addition, 83.9% of the infants were sensitized to at least one food allergen; 29% to at least one aero-allergen, which was comparable in both groups. For all infants, topical corticosteroids were prescribed before screening for this study. The topical corticosteroids were used in 93.5% of the infants for a mean (SD) of 124.6 (73.1) days before start of dietary intervention. Topical corticosteroid use was evenly distributed between the study groups, with a median (Q1–Q3) of 135 (67–150) days of topical steroid use in the control group and 113 (65–182) days in the active group, with no significant differences observed.

Low potency topical corticosteroids (hydrocortisone acetate 1%) were most commonly used (83.9%) compared with moderate potency triamcinolone acetonide 0.1% (29%) and high potency mometasone furoate 0.1% (9.7%).

The median [interquartile range (Q1–Q3)] for exclusive breastfeeding before study enrollment was 0 (0–3) months in the control group and 3 (0–6) months in the active group, but this was not significantly different. Cows’ milk protein was introduced at a median (Q1–Q3) of 0 (0–3) months and 3 (0–6) months in the control and active groups, respectively, (*p* > 0.05). Complementary feeding was first introduced at a median (Q1–Q3) of 5 (4–6) months among all infants.

### Effect on Severity of AD

At baseline, oSCORAD between the study groups was comparable, with a median (Q1–Q3) oSCORAD in the control group of 24 (21–27) and 21 (20–26) in the active group. After 4 months of dietary intervention, the average oSCORAD decreased in those infants (*n* = 26) who completed the study. The median change in both groups of −8, resulted in a median (Q1–Q3) post-intervention oSCORAD of 17 (12–21) in the control group and 13 (12–17) in the active group.

Interestingly, only four out of thirteen (31%) infants in the active group retained an oSCORAD above 15 (moderate AD) compared with eight out of thirteen (62%) infants in the control group. AD exacerbations occurred in 8 out of 26 infants (4 in both groups 30.8%) during dietary intervention period. The use of topical steroids during the dietary intervention period appears similar in both groups (*p* > 0.05) with a median (Q1–Q3) topical steroid use of 118.5 (113.5–120.8) days in the control group and 117.0 (113.0–121.5) days in the active group.

### Exploratory Immune Profiles in AD

**Figure 2:**
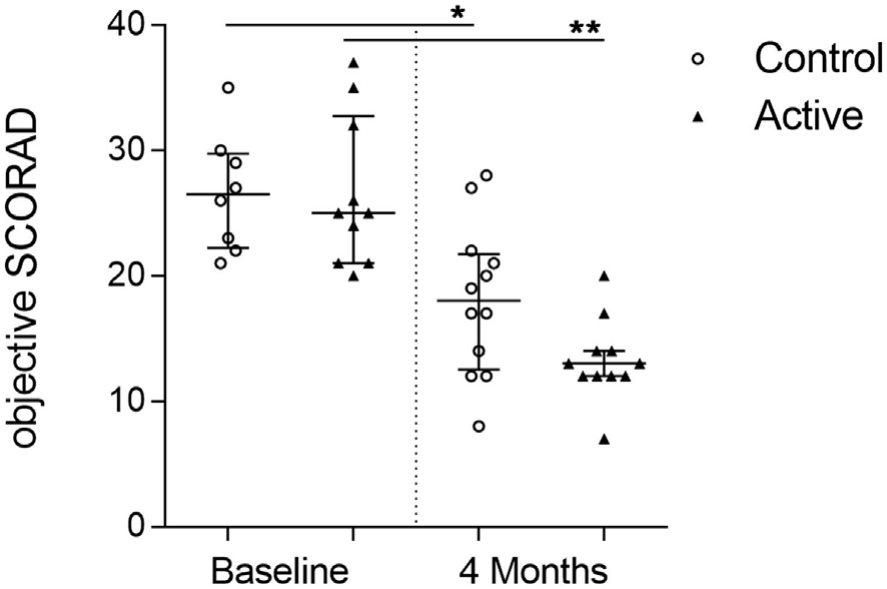
Reduction of atopic dermatitis severity over time. Individual oSCORAD of the control (open dots) and active (closed triangles) groups at baseline and after 4 months of dietary intervention, for infants from which serum samples were available (*n* = 41). Line represents median ± interquartile range. Significant differences within the groups are depicted with asterisks (non-paired W-test, *p < 0.05, **p < 0.01).

In total, 41 serum samples obtained from the clinical study were used for exploring the biomarker profiles of infants with moderate to severe AD and elevated IgE levels. For these infants from which serum samples were obtained, reduction of oSCORAD at baseline and after 4 months of dietary intervention is shown in (Figure 2). Non-parametric analyses were performed and indicated within the figures. Small sample sizes are in general a good argument to use non-parametric statistics, as possible skewness in the data may not be revealed. Additional parametric analysis using ordinary one-way ANOVA was performed, to show robustness of the data. The analysis indicated no differences between the groups at baseline and confirmed the significant decline within control (*p* < 0.001) as well as within active (*p* < 0.0001) of oSCORAD, which support the non-parametric analysis.

Several chemokines were measured in serum at baseline and at the end of intervention and correlated to AD severity. We detected an overall significant correlation between oSCORAD and CCL17 levels (Spearman *r* = 0.446, *p* < 0.01) and CCL22 (Spearman *r* = 0.288, *p* < 0.05) (Table 2). In addition, at baseline (*n* = 18), a significant correlation was detected between levels of IgE and chemokines CCL17 (Spearman *r* = 0.721, *p* < 0.01) and CCL22 (Spearman *r* = 0.549, *p* < 0.05). Moreover, a significant correlation between galectin-9 and chemokines CCL20 (Spearman *r* = 0.585, *p* < 0.05) and CCL22 (Spearman *r* = 0.492, *p* < 0.05) was observed.

**Table 2:** Two-tailed Spearman correlation of chemokines and T helper cell type (Th) 2/Th1 ratio to atopic dermatitis severity (oSCORAD) (*n* = 41).

Chemokine	Spearman *r*	*p* Value
CC chemokine ligand (CCL) 17	0.446	**0.002**
CCL20	0.163	0.273
CCL22	0.288	**0.049**
CXC chemokine ligand (CXCL) 9	−0.177	0.234
CXCL10	0.034	0.824
CXCL11	0.042	0.780
CCL17/CXCL9	0.375	**0.009**
CCL17/CXCL10	0.318	**0.029**
CCL17/CXCL11	0.364	**0.012**
CCL20/CXCL9	0.239	0.105
CCL20/CXCL10	0.032	0.832
CCL20/CXCL11	0.032	0.832
CCL22/CXCL9	0.259	**0.079**
CCL22/CXCL10	0.187	0.207
CCL22/CXCL11	0.187	0.208

**Figure 3:**
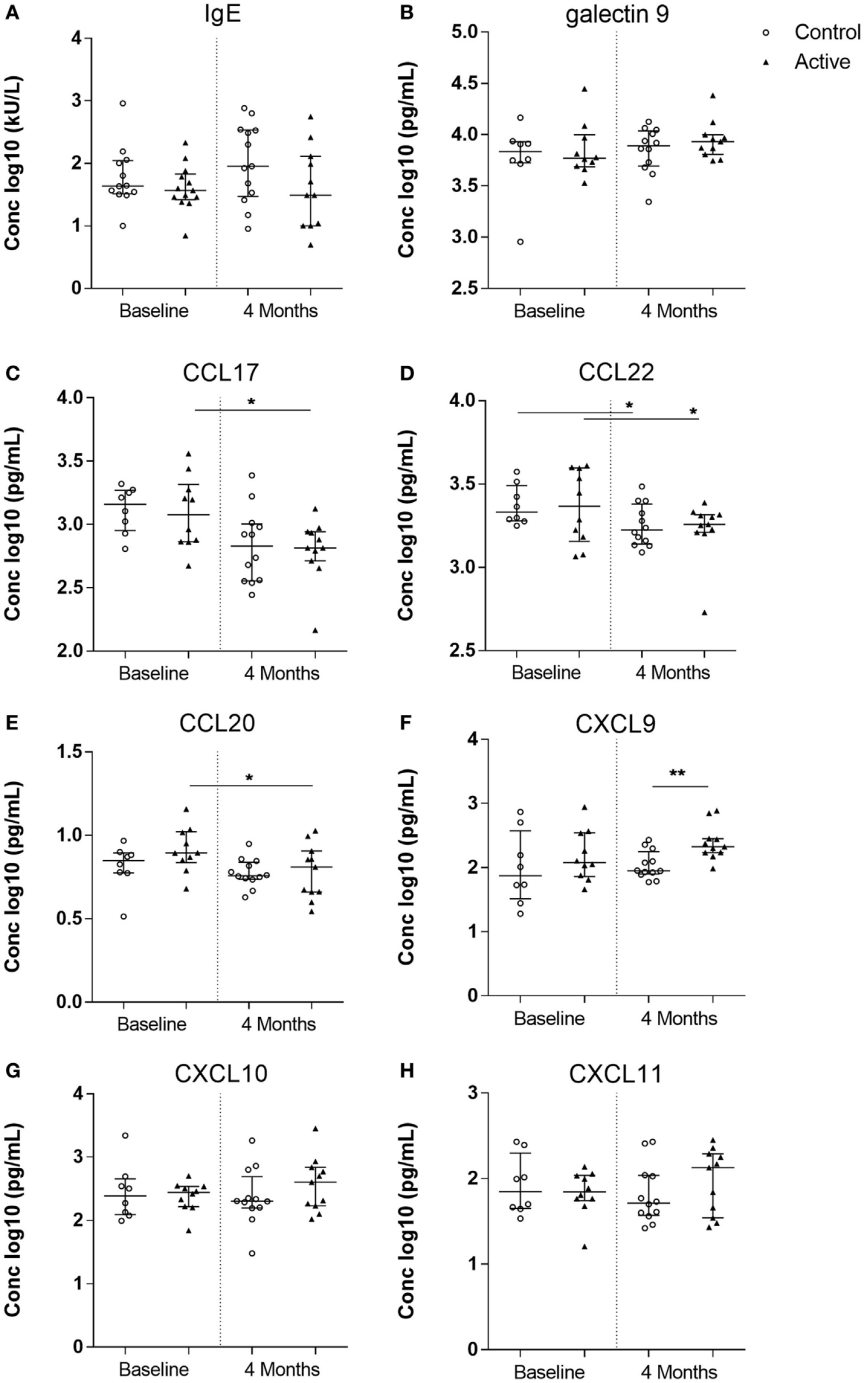
Allergy markers measured in serum of the control (open dots, *n* = 20) and active (closed triangles, *n* = 21) groups before and after dietary intervention. Immunoglobulin E (IgE) **(A)**, galectin-9 **(B)**, CC chemokine ligand (CCL) 17 **(C)**, CCL22 **(D)**, CCL20 **(E)**, CXC chemokine ligand (CXCL) 9 **(F)**, CXCL10 **(G)**, and CXCL11 **(H)**. Line represents median ± interquartile range. Significant differences within the groups are depicted with asterisks (non-paired W-test, *p < 0.05) and between groups (using Mann–Whitney *U*-test, **p < 0.01).

Total IgE and galectin-9 were measured in serum at baseline and at the end of intervention. Both levels, however, did not significantly change in either group (Figures 3A,B). The levels of Th2 driving chemokines CCL17 and CCL22 significantly changed over time (Figures 3C,D). In the control group, a significant reduction of CCL22, but not CCL17, was detected (W-test, *p* < 0.05), whereas in the active group, both CCL17 and CCL22 were significantly reduced (W-test, *p* < 0.05, for both). CCL20, a strong chemokine for DCs, remained unchanged in the control group, yet was significantly reduced in the active group after 4 months of dietary intervention (W-test, *p* < 0.05) (Figure 3E). While no differences were observed in the release of Th1-driving chemokines CXCL9, CXCL10, and CXCL11 in the control group, after 4 months of dietary intervention, CXCL9 levels were significantly higher in the active group as compared with the control group (MWU, *p* < 0.01). The dietary intervention resulted in a higher CXCL9 level (log 10 median, 2.33; range 1.99–2.89) in the active group as compared with the control group (1.95; 1.77–2.43) (Figures 3F–H). Moreover, additional parametric analysis using ordinary one-way ANOVA confirm our statistical findings within the serum biomarker analysis, although small sample size may not reveal possible skewness of the data and therefore the parametric analysis are not shown.

### Chemokine Ratios Change Over Time and due to Dietary Intervention

**Figure 4:**
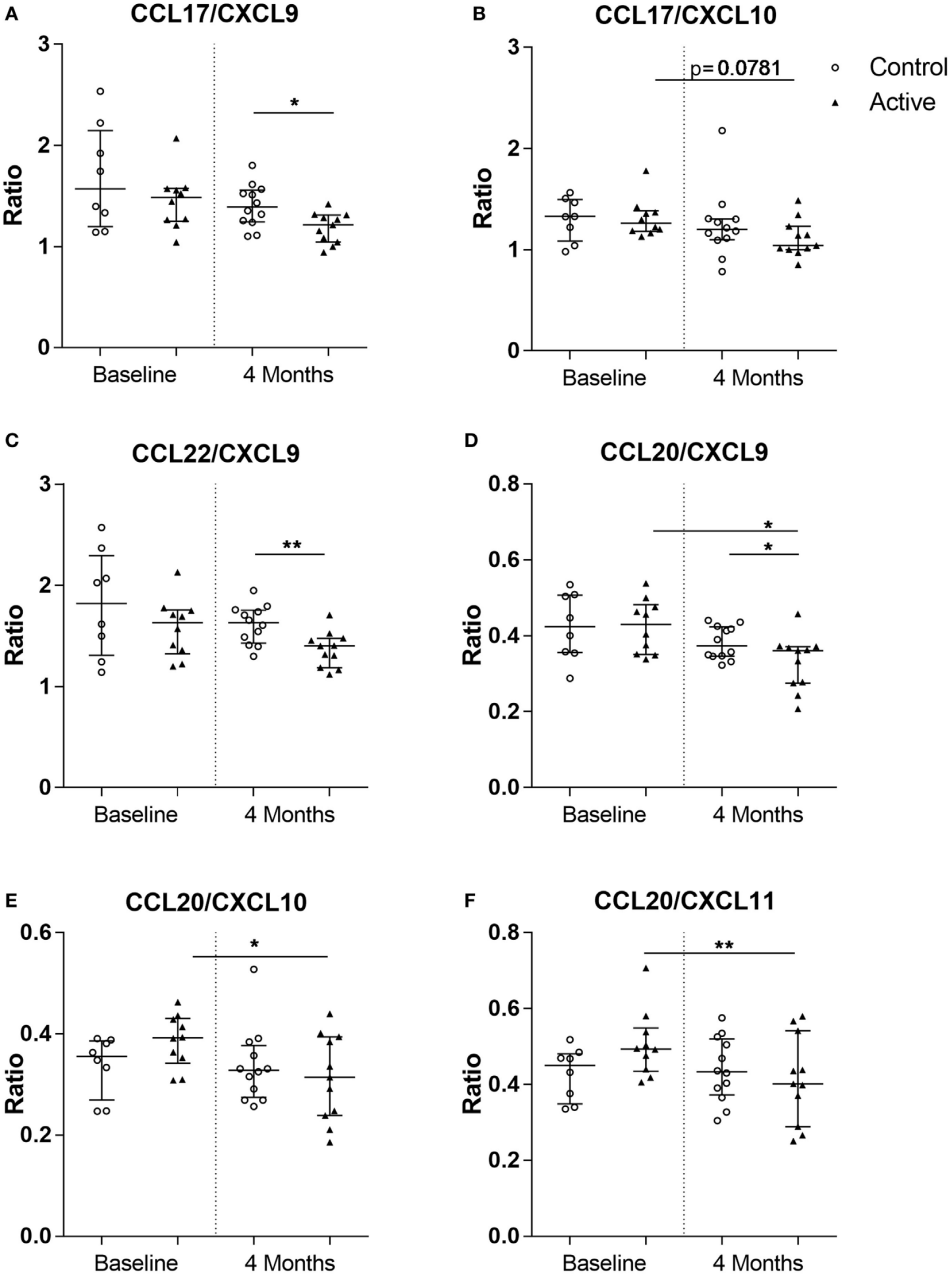
Chemokine ratios calculated in the control (open dots, *n* = 20) and active (closed triangles, *n* = 21) groups before and after dietary intervention. CC chemokine ligand (CCL) 17/CXC chemokine ligand (CXCL) 9 **(A)**, CCL17/CXCL10 **(B)**, CCL22/CXCL9 **(C)**, CCL20/CXCL9 **(D)**, CCL20/CXCL10 **(E)**, and CCL20/CXCL11 **(F)**. Line represents median ± interquartile range. Significant differences within the groups are depicted with asterisks (non-paired W-test, *p < 0.05, **p < 0.01) and between groups (using Mann–Whitney *U*-test, *p < 0.05, **p < 0.01).

Knowing that the immune balance between chemokines determines the type of cells recruited toward the lesional site, which in case of AD is the skin, nine chemokine ratios (Th2/Th1) were analyzed. After 4 months of dietary intervention, significantly lower CCL17/CXCL9 (MWU, *p* < 0.05), CCL20/CXCL9 (MWU, *p* < 0.05), and CCL22/CXCL9 (MWU, *p* < 0.01) ratios were observed in the active group as compared with the control group. Moreover, a reduction in CCL17/CXCL10 (W-test, *p* = 0.0781), CCL20/CXCL9 (W-test, *p* < 0.05), CCL20/CXCL10 (W-test, *p* < 0.05), and CCL20/CXCL11 (W-test, *p* < 0.01) ratios were observed over time within the active group. Interestingly, these ratios did not change in the control group over time (Figure 4).

## Discussion

Based on the hygiene hypothesis, the immunological basis in children with low microbial stimuli in early life is associated with higher Th2-immune responses (33, 34). Several studies have linked the infant microbiota of the gastrointestinal tract, as well as the skin and respiratory tract to the risk of developing childhood allergic diseases (35). This suggests that the microbiome plays an important role in early immunologic development (36). Several factors responsible for the microbial dysbiosis associated with allergy development are cesarean delivery (37), lack of breast milk (38), and antibiotics use in early life (39). Specific dietary interventions have been shown to alter microbial development and the immune system by changing the Th2/Th1 balance (17, 18). An immunological imbalance may activate the secretion of IL4, IL5, and IL13 production, which may be involved in the induction of IgE and eosinophil activation, leading to development of allergic manifestations (14). In this exploratory setting, a study with specific dietary intervention in infants with moderate-to-severe AD and elevated IgE levels was used to investigate changes in chemokine profiles. The changes in chemokine ratios within infants receiving dietary intervention suggest a specific change in immune response where the Th2/Th1 balance skews away from the allergic phenotype.

It has previously been reported that dietary intervention with scGOS/lcFOS plus *B. breve* M-16V in infants with AD and elevated IgE levels resulted in a significant improvement in AD severity (19), eventually leading to the prevention of asthma-like symptoms later in life (20). In this study, infants presenting with AD and elevated IgE levels were included to investigate the AD pathology within this unique population. While we did not find a direct influence on total IgE levels, galectin-9 tended to increase after dietary intervention, especially in the active group, although this did not reach statistical significance. The time frame and size of this study may have been too short and too small to observe any potential differences in IgE. However, changes in serum chemokine profiles including, CCL17, CCL20, CCL22, CXCL9, CXCL10, and CXCL11 were detected.

In early AD, immune reactivity is skewed toward a Th2-type response; during the acute phase keratinocytes, DCs and dermal fibroblasts produce CCL17 and CCL22, thereby recruiting Th2 cells *via* CCR4 (6, 40, 41). Moreover, IFNγ-induced Th1-associated CXCR3 ligands CXCL9, CXCL10, and CXCL11 can be measured in AD patients (42–44) and are known to play a role in the chronic phase of AD (45, 46). Furthermore, while excess Th2 activation characterizes AD in both children and adults, Th17 polarization is also highly activated at disease initiation in pediatric AD. The inflammatory chemokine CCL20 was previously observed to be significantly higher in the lesions of the skin of children younger than 5 years of age as compared with adults (8, 12). In this study, decreased levels of Th2-driving chemokines CCL17 and CCL22 and inflammatory chemokine CCL20 were detected, while the level of Th1-driving chemokine CXCL9–11 changed after 4 months of dietary intervention. These results suggest that dietary intervention with prebiotics scGOS/lcFOS and a probiotic *B. breve* skews the allergic phenotype from a Th2-dominant toward a Th1-type immune response. In early life, immune skewing toward Th1 responsiveness is essential and is actively present (8). It has been reported that high Th2-associated CCL22 and low Th1-associated CXCL10 levels in cord blood (including CCL22/CXCL10) precedes IgE-mediated allergic sensitization in early life (47). Moreover, high cord blood levels of CCL17 and CCL22 were shown to precede allergy development during the first 6 years of life (48). Within our study, we detected significant positive association between IgE and CCL17, IgE and CCL22, galectin-9 and CCL20, and galectin-9 and CCL22 at baseline. Of these, CCL17 is a commonly used biomarker for AD severity, primarily in adults (13, 49). We also observed that CCL17 was significantly associated with disease severity (oSCORAD) in infants. Although age in itself may affect the development of AD, within our study, specifically the Th2/Th1 chemokine ratios were reduced after 4 months of dietary intervention in the active group, as can be seen in (Figure 4). This is also suggestive for the hypothesis that the changes (decrease) in chemokine level precede changes in IgE level in association of changes in AD severity. Although correlation analysis does not establish a cause-and-effect relationship, we show associations of serum biomarkers, ratios and changes herein to changes observed in AD severity (oSCORAD).

This study has several limitations. The specific requirements for study inclusion limited recruitment and therefore only a small sample size was enrolled. However, the uniqueness of the included group did allow us to explore associations between AD severity and chemokine profiles in this population of young infants with AD and elevated IgE levels. Heterogeneity between studies with dietary interventions are high, and therefore focused clinical randomized controlled trials are needed to understand the potential role and underlying mechanism of these dietary interventions in children with AD (50). As it is unethical to withhold infants with AD this treatment, the use of topical steroids was permitted during the intervention period when the topical steroids where already prescribed before start of intervention. However, topical corticosteroids were equally distributed in both groups at baseline (Table 1) and their use during the dietary intervention period appears similar in both groups, with a median of 118.5 days (control), and 117 days (active) with no significant differences. Local corticosteroid use was therefore not included as a confounding variable. In total, six infants received oral antibiotics for adverse events including fever and/or respiratory tract infections during the dietary intervention. As the antibiotic use was equally distributed in both groups (three infants from each group), these infants were not excluded from the serum analyses. Due to challenges with obtaining blood samples from infants, at both time points there were missing blood samples or not enough blood for serum analyses due to priority of total and/or specific IgE analyses because it was an inclusion criteria to have elevated total or specific IgE levels. Therefore, only 41 serum samples obtained from the clinical study were used in this current study to explore biomarker profiles in these AD infants. Despite this limited number of samples, significant changes and associations were observed in Th2- and Th1-chemokine profiles. In addition, due to the exploratory nature of this study, correction for multiple testing was not conducted. Small sample sizes are in general a good argument to use non-parametric statistics, as possible skewness in the data may not be revealed. The additional parametric analysis showed robustness of the data, although confirmation of our results in larger cohorts is needed.

In conclusion, this study provides interesting insights into the complex pathology of AD and suggests that chemokine profiles and ratios may be useful for detecting a change in allergic manifestation. Furthermore, dietary intervention in infants with AD was associated with an improvement in Th2/Th1 balances, skewing away from the allergic phenotype.

## The Clinical Study Group

The following authors, who are listed in alphabetical order, contributed to the work of the Clinical Study Group: **Blauw A.**, **Dontje B.**, and **Duijvestein Y. C. M.**, Department of Paediatrics, MCA Alkmaar, Netherlands; **de Boom W. H. C.** and **Groot I.**, Department of Paediatrics, Waterland Hospital Purmerend, Netherlands; **Boks M.** and **van Kooyk Y.**, Department of Molecular Cell Biology and Immunology, VUMC Amsterdam, Netherlands; **Fandri D. H. H.**, Department of Paediatrics, St. Antonius Nieuwegein, Netherlands; **Hijnen D.**, Department of Dermatology, UMCU Utrecht, Netherlands; **Middelkamp-Hup M. A.**, Department of Dermatology, AMC Amsterdam, Netherlands; **Papi B.**, **Roelofs M.**, **Rijnierse A.**, **Veening D.**, and **Clinical Trial Support**, Nutricia Research, Utrecht, Netherlands; **Prakken B. J.**, University Medical Centre Utrecht, Department of Paediatric Immunology, Laboratory of Translational Immunology, Utrecht, Netherlands; **Ten Tusscher G. W.**, Department of Paediatrics, WFGH Hoorn, Netherlands; **Tupker R. A.**, Department of Dermatology, St. Antonius Nieuwegein, Netherlands; **Willemsen L. E. M.**, Faculty of Science, Utrecht Institute for Pharmaceutical Sciences, Utrecht University, Utrecht, Netherlands.

## Ethical Statement

This trial was approved by the Medical Ethics Review Committee of the AMC Amsterdam (METC 2012_032) and is registered in the trial register (NTR number NTR3447). Written informed consent was obtained from parents/legal guardians of all infants.

## Author Contributions

Performed the study; LH, DB, SO, AS, and the Clinical Study group. Designed and supervised the study; DB, JG, LK, ES, WA, AS, and BL. Participated in data collection, analyses, and interpretation; LH, SO, AW, MC, WJ, and BL. Drafted the manuscript LH, SO, AW, BL, and AS. All the authors revised and approved the final version of the manuscript.

## Conflict of Interest Statement

JG is head of the Division of Pharmacology, Utrecht Institute for Pharmaceutical Sciences, Faculty of Science at the Utrecht University and partly employed by Nutricia Research. LK and BL, as indicated by the affiliations, are part of a strategic alliance between Utrecht University, University Medical Centre Utrecht and Nutricia Research and are employed by Nutricia research. WJ is sponsored by Understanding Childhood Arthritis Network (UCAN) and Technology Foundation STW (Stichting voor de Technische Wetenschappen). All other coauthors declare no potential conflict of interest.

## Abbreviations

Active group, group of infants receiving the whey based extensively hydrolysed infant formula with short-chain galacto-oligosaccharides, long-chain fructo-oligosaccharides and *Bifidobacterium breve* M-16V; AD, atopic dermatitis; AMC, Academic Medical Centre, Amsterdam, the Netherlands; CCL, CC chemokine ligand (-17, -20, -22); Control group, group of infants receiving the whey based extensively hydrolysed infant formula; CXCL, CXC chemokine ligand (-9, -10, -11); DC, dendritic cell; HOME, Harmonising Outcome Measures for Eczema; IgE, immunoglobulin E (total, specific); IL, interleukin (-4, -5, -13, -22, -31); IFN, interferon (−γ); lcFOS, long-chain fructo-oligosaccharides; IQR, interquartile range (Q1–Q3); MWU, Mann–Whitney *U*-test; oSCORAD, objective SCORing Atopic Dermatitis score; scGOS, short-chain galacto-oligosaccharides; SCORAD Score, scoring atopic dermatitis score, clinical tool for scoring AD severity; Th, T helper cell type (1, 2, 17, 22); W-test, Wilcoxon matched-pairs signed rank test.
